# Obesity Characteristics Are Poor Predictors of Genetic Mutations Associated with Obesity

**DOI:** 10.3390/jcm12196396

**Published:** 2023-10-07

**Authors:** Ahmed W. Al-Humadi, Khaled Alabduljabbar, Moath S. Alsaqaaby, Hani Talaee, Carel W. le Roux

**Affiliations:** 1 Diabetes Complications Research Centre, Conway Institute, University College Dublin, D04V1W8 Dublin, Ireland; a.alhumadi@svph.ie (A.W.A.-H.); khalabduljabbar@kfshrc.edu.sa (K.A.); malsaqaaby@kfmc.med.sa (M.S.A.); htalaee@svhg.ie (H.T.); 2 Department of Dentistry, Hilla University College, Babylon 510001, Iraq; 3 Department of Family Medicine and Polyclinics, King Faisal Specialist Hospital and Research Centre, Riyadh 11211, Saudi Arabia; 4 Obesity, Endocrine and Metabolism Centre, King Fahad Medical City, Riyadh 11525, Saudi Arabia

**Keywords:** genetic mutations, genetic obesity, leptin–melanocortin pathway, obesity, POMC

## Abstract

Background: The genetic contribution to obesity is substantial and may underpin the altered pathophysiology. One such pathway involves melanocortin signaling in the hypothalamus. Genetic variants can cause dysregulation in the central melanocortin pathway that can result in early onset of hyperphagia and obesity. Clinically identifying patients who are at risk of known genetic mutations is challenging. The main purpose of this study was to identify associations between the clinico-demographical characteristics and the presence of a genetic mutation associated with obesity. Methods: We tested samples from 238 adult patients with class III obesity between October 2021 to February 2023 using next-generation sequencing (NGS) (Illumina, NovaSeq 6000 Sequencing System). The results were classified as “no variant identified” or “variant identified”. Results: 107 patients (45%) had one or more gene mutation in the leptin–melanocortin pathway. All variants were heterozygous. The patients with a gene mutation had a BMI of 48.4 ± 0.8 kg/m^2^ (mean ± SEM), and those without a gene mutation had a BMI of 49.4 ± 0.7 kg/m^2^ (*p* = 0.4). The mean age of onset of obesity in patients with a gene mutation was 13.9 ± 1.3 years and for those without gene mutations was 11.5 ± 0.9 years (*p* = 0.1). The incidence of hyperphagia as a child was also not predictive (*p* = 0.4). Conclusions: Gene mutations associated with obesity in patients with a BMI > 40 kg/m^2^ are common. However, a patient’s BMI, age of onset of obesity, or age of onset of hyperphagia did not help to differentiate which patients may be more likely to have genetic mutations associated with obesity.

## 1. Introduction

Obesity is increasing worldwide and is now considered a complex and chronic multifactorial disease that is defined as excess or abnormal adipose tissue causing a deterioration in health, which includes long-term health complications and excess mortality [1,2,3,4,5]. Estimates of genetic heritability of obesity range from 40% up to 75% [6]. The melanocortin pathway in the hypothalamus is the best example of a key pathway that plays a fundamental role in weight regulation in humans and animals [7,8]. Genetic variants cause dysregulation in the central melanocortin pathway that results in early onset of hyperphagia and obesity [7,8].

Monogenic obesity is rare; it happens in a Mendelian pattern and entails single-gene defects or small/large chromosomal deletions, resulting in an early-onset and severe form of obesity in children [9]. Conversely, polygenic obesity is more common; it also follows a heritability pattern and entails the occurrence of several polymorphisms that collectively exert combinatorial obesity-promoting effects, resulting in body fat accumulation in adolescents and adults [9].

Early characterization of a person’s genetic predisposition to obesity has several benefits, including a diagnosis of the subtype of obesity, identification of novel molecular targets for anti-obesity therapies, and facilitation of other treatments [10,11]. Several studies attempted to describe the frequency of common and rare disease-causing genetic variants of obesity [12,13,14,15].

Since individuals with BMI ≥ 30 kg/m^2^ have remarkable heterogeneity, defining obesity relying on BMI has been considered too complex to be a homogeneous entity [16]. The risk of obesity differs upon factors such as regional fat distribution, cardiorespiratory fitness, the quality of feeding, and level of physical activity [17]. Therefore, populations with obesity are classified as a lower-risk subgroup if it comprises individuals who have low levels of visceral adipose tissue, eat well, and are physically active despite being obese at any BMI grade [18]. At the other end of the obesity spectrum, the high-risk subgroup comprises individuals who have high levels of visceral adiposity and features of metabolic syndrome, poor dietary patterns, and a sedentary lifestyle, putting them at high risk for poor health outcomes, particularly cardiovascular events [16,19].

Obesity can be classified into several categories based on the body mass index (BMI) [20], of which class III obesity (severe obesity) is defined as individuals having a BMI ≥ 40 kg/m^2^ [20]. The purpose of this single-center, observational study was threefold: (i) to explore the clinico-demographical profile of individuals with class III obesity, (ii) to determine the prevalence of the common and rare disease-causing genetic variants of obesity, and (iii) to examine the association between the clinico-demographical characteristics and the presence of a genetic mutation associated with obesity.

## 2. Materials and Methods

An observational single-center study was performed according to the Declaration of Helsinki and the local Research Ethical Committee for research studies in St. Vincent’s University Hospital. Patients were recruited from a specialist obesity service where patients were referred for the treatment of obesity. A total of 238 adult (female/male) patients were tested between October 2021 to February 2023 in the Obesity Complications Clinic at St Vincent’s University Hospital, Dublin, Ireland.

All patients were provided information about genetic testing for obesity, and they provided their written informed consents. Patients with obesity having a BMI ≥ 40 kg/m^2^ were included. History of onset of obesity and age of hyperphagia, excessive eating over a prolonged time, were recorded. To document the history of hyperphagia, patients were asked whether they remembered feeling hungry as a child and whether their family members commented that they “had a very good appetite” as a child. The body weight and height were measured using digital scales (Marsden Weighing Machines Group Ltd., Rotherham, UK) with precision of 0.1 kg. The highest weight was reported either in the clinic notes or as recalled by the patient.

Data on failure to thrive before age of two years old, thyroid pathology, prior antiobesity medications, bariatric surgery, family history of obesity, and family history of inherited disease and/or earlier genetic testing were collected. Patients were clinically assessed if they had characteristics of Bardet Biedl Syndrome (BBS).

Rhythm Pharmaceuticals Inc. (Boston, MA, USA) supported the logistics and cost of the genetic testing as part of their Rare Obesity Advanced Diagnosis (ROAD) program [21,22]. Venous whole blood samples were collected and preserved immediately in EDTA tubes, and analysis was performed by Unilabs, Porto, Portugal. The results were classified as negative (no variant identified) or positive (variant identified) based on predefined genes list of the Rare Obesity Advanced Diagnosis (ROAD) program [23]. The positive results were categorized according to American College of Medical Genetic guidelines, and the variant mutations were classified as follows: benign, likely benign, uncertain significance, likely pathogenic, and pathogenic [24].

The framework utilized to select the 80 genes tested in this panel was based on a set of clinical (human genetics) and non-clinical experimental evidence to evaluate MC4R pathway relevance, and this framework is based on the core principles of the NIH ClinGen Gene-disease Clinical Validation approach and is the industry standard for assessing gene–disease relationships. Thus, human genetic evidence helped define the contribution of the gene to human obesity, while experimental evidence assessed the involvement of the gene in the function of the MC4R pathway. The cumulative evidence was then used to classify the MC4R pathway’s genes into four strength-based tiers: very strong, strong, moderate, and weak. The nature, quantity, and quality of evidence required for each tier was built upon that of the previous tier, with higher-ranked genes being most likely to define patient populations that could be potentially responsive to long-term setmelanotide treatment. Based on a comprehensive literature review, 80 MC4R pathway genes were identified, ranked, and ordered: 8 “very strong”, 29 “strong”, 22 “moderate”, and 21 “weak” [25,26].

### 2.1. DNA Analysis

Next-generation sequencing (NGS) (Illumina, NovaSeq 6000 Sequencing System, San Diego, CA, USA) of genomic DNA was performed upon capture of target regions of the whole exome using oligonucleotide probes (Human All Exon V6, Agilent Technologies, Santa Clara, CA, USA). The NGS data analysis was executed after alignment, base/CNV (copy number variations) calling, and annotation (Illumina, Base Space, DragenV3.7.5) using as reference the homo sapien genome (UCSC hg19), and SNVs (single nucleotide variants), indels, and CNVs were filtered, and a structured analysis (Alissa software, https://www.allisa.software/ (accessed on 21 August 2023), Agilent Technologies) was performed in order to assess their pathogenicity and potential to explain the clinical phenotype. The variants detected in the flanking exons and intronic regions (±8 bp) of the genes were evaluated. Variant classification and description were performed according to international recommendations [24,27,28,29]. SNVs, indels, and CNVs that confirm or most likely explain the phenotype are reported.

Variants reported in the result that do not meet the quality control criteria (depth detected in the test), for which the gathered evidence does not conclusively support a causal effect for the indicated phenotype was also be reported (e.g., partial phenotypic overlap, inconclusive pathogenicity criteria, and variant of a gene with unknown or poorly characterized clinical association). Pathogenic and probably pathogenic variants were reported; variants of unknown clinical significance were reported only if that variant had been identified in an index case or if the phenotype was highly suggestive for the disease in the study; benign and probably benign variants and variants of unknown clinical significance that do not seem to explain the disease in the study were not reported. Variant classification may change over time, due to novel population, scientific, or clinical data. It cannot be excluded that pseudogene sequences, highly homologous sequences, repetitive sequences, or regions with lower coverage may interfere with the technical ability to identify SNVs, indels or CNVs in the test. The analysis did not exclude variants outside of the analyzed regions or not detected by this methodology (e.g., triplet repeats expansions, epigenetic modifications, and variants with low-level mosaicism). CNV analysis was the screening method used, and the number of targets, size, and nature of the involved regions influenced the sensitivity and specificity of CNV calling. The detection of CNV with two (or more) exons/targets was more sensitive, although equally specific (CNVs detection sensitivity/specificity involving two or more targets 94.4%/99.3%; CNVs detection sensitivity/specificity involving one or more targets 87.5%/99.3%). In the low-quality samples (with highly degraded DNA), lower quality NGS data was generated; CNV analysis was not performed. The result and interpretation are dependent upon proper identification of the received sample and the provided clinical information.

Secondary analysis included creating three categories: category 1 with genetic variants that have been approved for treatment with the melanocortin-4 receptor (MC4R) agonist, setmelanotide, in Europe, as well as those under investigation for setmelanotide efficacy in clinical trials (Table S1); category 2 with genetic variants that may contribute to a diagnosis of genetic obesity but are neither currently indicated for setmelanotide treatment nor under investigation for setmelanotide efficacy in clinical trials (Table S1); and category 3 without any gene mutations.

### 2.2. Statistical Analysis

Statistical analysis was performed using IBM SPSS software version 28.0 (IMB, Armonk, NY, USA). For continuous variables, descriptive statistics were initially examined for normality using graphical methods such as histograms and Q-Q plots along with analytical methods including the Kolmogorov–Smirnov test. In instances where data were normally distributed, the results were presented as mean and standard error of the mean (SEM). Conversely, for non-normally distributed data, median and interquartile range (IQR) were reported. Categorical variables were reported as frequencies and percentages. Chi-square test, *t*-test, and ANOVA, as appropriate, were applied to evaluate the differences between the groups. The significance level for all analyses was maintained at *p* ≤ 0.05.

## 3. Results

**Figure 1:**
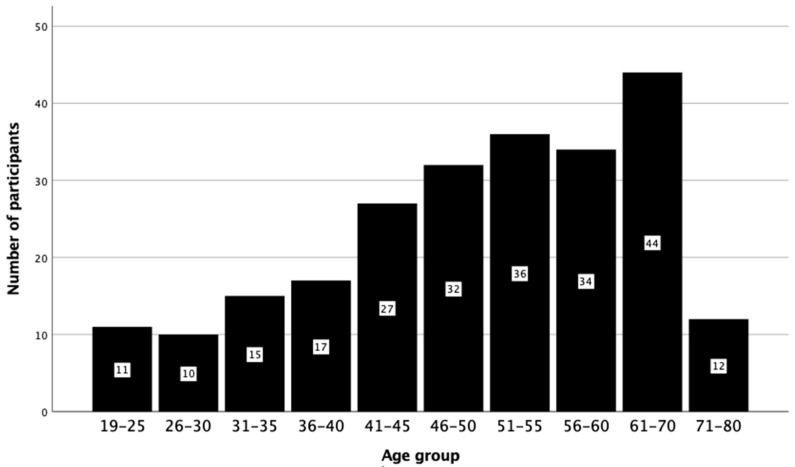
The distribution of participants according to age groups.

A total of 238 adults with a BMI > 40 kg/m^2^ including 89 males (37.4%) and 149 females (62.6%) were tested. The ages ranged from 19 to 77 years, with a median (IQR) of 51 (18) years (Table 1). Figure 1 shows the distribution of ages in the cohort. None of the participants had syndromic features on examination.

### 3.1. Heaviest Weight in Lifetime and Weight at Screening

Several patients were undergoing treatment for obesity, and thus, the heaviest weight of patients and their weights at the start of screening were recorded. The mean of the heaviest weight was 140.2 ± 1.8 kg, while the mean of weight at the screening was 119 ± 1.9 kg. The mean of the heaviest BMI was 48.9 ± 0.5 kg/m^2^, while the mean of BMI at screening was 41.3 ± 0.6 kg/m^2^ (Table 1).

### 3.2. Age of Hyperphagia, Onset of Heaviest Weight, and Onset of Obesity

The onset age of hyperphagia ranged between 4 months and 43 years, with a mean of 7.1 ± 0.4 years and a median of 6 years. While the mean age of onset of obesity was 12.6 ± 0.8 years. The mean age when the heaviest lifetime weight was reached was 42.5 ± 0.8 years (Table 1).

Table 1 also shows a group of descriptive indicators related to the participants’ medical and family histories. Overall, 71.8% of the participants had a history of childhood obesity. History of hyperphagia was reported by 194 individuals (81.5%). History of obesity in a family member was reported by 78.6% of participants.

### 3.3. Genetic Test Results

Among 238 individuals, 107 patients tested positive for genetic mutation (45%) (Table 2). Furthermore, 111 unique variants were reported in 47 different genes. A single gene variant was found in 81 patients (34%), two genes were found in 19 patients (8%), while three gene variants were reported in 6 patients, and 1 patient was found to have four gene variants. All variants were heterozygous, and two of them were reported as hemizygous. The findings were subdivided into three categories: category 1 encompasses 83 patients (34.9%) with genetic variants that have been approved for treatment with the MC4R agonist setmelanotide in Europe, as well as those under investigation for setmelanotide efficacy in clinical trials (Table S1); category 2 includes 24 patients (10.1%) with genetic variants that may contribute to a diagnosis of genetic obesity but are neither currently indicated for setmelanotide treatment nor under investigation for setmelanotide efficacy in clinical trials (Table S1); and category 3 consists of 131 patients (55%) without any gene mutations (Table 2). Genes panel and the risk assessment in regard to obesity are reported in Table S2. Overall, 85 of the 107 patients (79.4%) with detected gene mutations were classified as VUS (genetic variant of uncertain significance). The prevalence of each genetic variant of uncertain significance among the positive cases are presented in Table S3.

The differences between the two groups, positive and negative, in genetic testing were assessed using independent sample *t*-test for continuous variables and Chi-square test for categorical variables (Table 3). The results were subdivided into different subgroups, and comparisons were made between the presence or absence of these phenotypical features and the frequency for the mutations we screened for. When examining the data according to above or below the median for heaviest lifetime BMI (kg/m^2^), heaviest lifetime weight (kg), onset of obesity (years), and age of hyperphagia (years) or examining the data according to positive or negative history of childhood obesity, positive or negative history of hyperphagia during childhood, and positive or negative family history of obesity, we found no difference in the frequency of mutations. There were no significant differences in clinical features between the two groups who had positive and negative results for the genetic test. The patients were divided into the three categories as mentioned above, and the clinical characteristics of the three groups were not significantly different (Table 4).

## 4. Discussion

All the patients were from the Dublin region in Ireland, and they were all classified as white Irish. The Republic of Ireland has a very homogenous society, and hence, there were no major differences in the population we studied compared to the background population who live in the county of Dublin. The patients tested were not treatment naïve, with several having had pharmacotherapy or bariatric surgery. Thus, at the time of the genetic test, the burden of complications was not a true reflection of the impact of obesity.

Almost half of patients that we tested had a heterozygous gene mutation associated with obesity. We could not identify clinical features that could have helped us to predict whether a patient may have been more likely to have one of the genetic mutations that we screened for. More than 70% of all our patients had a history of childhood obesity, hyperphagia as a child, and a family history of obesity [9,30,31]. We found no association between genetic mutations detected and various clinico-demographical characteristics in adult population with class III obesity.

Genetic disruptions involving the hypothalamic leptin–melanocortin signaling pathway account for the vast bulk of genetic predispositions to obesity. Most notably, genetic variants involving *PCSK1*, *POMC*, *LEP*, *LEPR*, and *MC4R* genes are the most frequently reported events [32]. We found 21 genetic variants (8.8%) affecting *PCSK1* consistent with a large-scale meta-analysis [33]. We also detected eight genetic variants affecting POMC (3.4%) that have been implicated in the development of obesity [34,35]. We did not identify any variants involving leptin signaling (LEP and LEPR).

All genetic variants in our research subjects were heterozygous. Courbage et al. [36] conducted a population-based study on 1486 subjects with severe obesity, which included 600 children and 886 adults. The genes of interest in their research comprised *PCSK1*, *POMC*, *LEP*, and *LEPR*. The rate of patients with heterozygous variants was higher compared with those with homozygous variants (roughly 7% vs. 2%, respectively). However, adults with homozygous variants had higher BMIs, earlier onset of obesity, augmented food impulsivity, and worse hormonal derangements compared with adult patients harboring heterozygous variants. Overall, the study concluded that heterozygous variants involving *PCSK1*, *POMC*, *LEP*, and *LEPR* are common among adult patients with class III obesity and occasionally may exhibit a phenotype similar to that of homozygotes.

Early detection of genetic mutations can help diagnose the subtype of obesity, identify novel molecular targets for anti-obesity drugs, and facilitate the start of treatment [37]. Setmelanotide is an effective medication for the disease of obesity in patients older than 6 years if the patient has *POMC* deficiency, *PCSK1* deficiency, *LEPR* deficiency, or Bardet–Biedl syndrome [21,38,39,40].

Despite the technical, psychological, and behavioral challenges, genetic testing for obesity could be an essential healthcare practice for patients who are at high risk of gaining more weight in the future [9,41].

Our study has several limitations. The small sample size is atypical compred to large consortiums conducting genetic studies in the field, but the numbers are relatively high for the clinic who may start conducting routine accompanying genetic testing to determine eligibility for medications such as setmelanotide. Furthermore, the lack of a control arm made subjective the background prevalence of the observed variants in the population from which the patients were derived as well as some information regarding the height and failure to thrive. The genetic panel employed in the current study might not be comprehensive enough to capture all the potential common and rare disease-causing genetic variants of obesity, but the panel was specific for deciding eligibility for the treatment and future research studies with setmelanotide. Furthermore, our analysis did not take into consideration the correlation between the severity of the BMI and the likelihood of having a positive genetic testing. We appreciate that heterozygous mutations in genes such as *ALMS1*, *BBSs*, *LEPR*, *POMC*, and *PCSK1* have not been shown to be sufficient to cause the disease of obesity, except for the complete loss-of-function mutations of *PCSK1* [42], but not for *POMC* or *LEPR* mutations [43]. The heterozygous mutation NM_198428.3:c.(442+1_443-1)_(702+1_703-1)del in the BBS9 gene may not be considered pathogenic because the gene has only been shown to be involved in monogenic obesity in a recessive model. Thus, a major limitation is that the heterozygous nature of the majority of these mutations has not been implicated in obesity. The American College of Medical Genetics and Genomics (ACMG) criteria for each mutation has not been provided, but rather we used the classification that would allow patients to enter into clinical studies or treatment with setmelanotide. Given our relatively small number of patients, we were not able to adjust for age, sex, and ancestry.

## 5. Conclusions

Gene mutations for obesity in patients with a BMI > 40 kg/m^2^ are common; however, the medical history of patients with and without gene mutations appear similar. This makes it difficult to predict which patients will have a genetic mutation associated with obesity prior to testing. The recommendation of our findings is that obesity clinicians who are not genetic specialists should not be discouraged when they start genetic testing and do not find a large number of actionable gene mutations linked with obesity. Features such as early onset hyperphagia and early onset obesity are recognized as helpful, but the effect size of the predictive value of these features is limited.

## Figures and Tables

**Table 1:** Characteristics and descriptive indicators related to the participants’ health parameters and medical and family histories (*n* = 238).

Characteristics/Indicator	Categories	*n*	Median */Mean	Percentage/IQR */SEM
Gender	Male	89		37.4%
	Female	149		62.6%
Age (years)		238	51 *	18 *
Height (cm)		238	169.2	0.6
Heaviest lifetime weight (kg)		238	140.2	1.8
Weight at screening (kg)		238	119	1.9
Heaviest lifetime BMI (kg/m^2^)		238	48.9	0.5
BMI at screening (kg/m^2^)		238	41.3	0.6
Onset of hyperphagia (years)		238	7.1	0.4
Age of heaviest weight (years)		238	42.5	0.8
Onset of obesity (years)		238	12.6	0.8
History of childhood obesity	Yes	171		71.8%
	No	67		28.2%
History of hyperphagia	Yes	194		81.5%
	No	44		18.5%
History of failure to thrive before the age of 2	Yes	13		5.5%
	No	222		93.3%
	Unknown	3		1.3%
History of thyroid disease	Yes	37		15.5%
	No	199		83.6%%
	Unknown	2		0.8%
History of using anti-obesity medication	Yes	151		63.4%
	No	87		36.6%
History of bariatric surgery	Yes	83		34.9%
	No	155		65.1%
Family history of obesity	Yes	187		78.6%
	No	42		17.6%
	Unknown	9		3.8%
	Family history of obesity (Father)	97		40.8%
	Family history of obesity (Mother)	113		47.5%
	Family history of obesity (Siblings)	129		54.2%

*n*: number; SEM: standard error of the means; and IQR: interquartile range.

**Table 2:** Genetic test results and categories (*n* = 238).

Outcome	Categories	*n*	%
Genetic test result	Negative	131	55
	Positive	107	45
Number of subjects with gene mutations detected	1-gene mutation	81	34
	2-gene mutations	19	8
	3-gene mutations	6	2.5
	4-gene mutations	1	0.4
Gene category ^a^	Category 1	83	34.9
	Category 2	24	10.1
	Category 3	131	55

*n*: number; ^a^ category 1: genetic variants that have been approved for treatment with the melancortine-4 receptor (MC4R) agonist setmelanotide in Europe, as well as those under investigation for setmelanotide efficacy in clinical trials; category 2: genetic variants that may contribute to a diagnosis of genetic obesity but not a part of category 1; and category 3: no gene mutations.

**Table 3:** Analysis of different variables in relation to the genetic test results.

Variable	Genetic Result	Mean/Frequency	SEM or Percentage	*p* Value
Heaviest lifetime BMI (kg/m^2^)	Negative	49.4	0.7	0.4
	Positive	48.4	0.8	
Heaviest lifetime weight (kg)	Negative	140.9	2.3	0.7
	Positive	139.4	2.7	
Onset of obesity (years)	Negative	11.5	0.9	0.1
	Positive	13.9	1.3	
Age of hyperphagia (years)	Negative	6.9	0.4	0.4
	Positive	7.5	0.6	
Positive history of childhood obesity	Negative	99	41.6%	0.2
	Positive	72	30.3%	
Positive history of hyperphagia during childhood	Negative	104	43.7%	0.4
	Positive	90	37.8%	
Positive family history of obesity	Negative	101	42.4%	0.7
	Positive	86	36.1%	

SEM: standard error of the mean.

**Table 4:** Analysis of different variables in relation to the subgroups according to gene category ^1^.

Variable	Gene Category	Mean/Frequency	SEM or Percentage	*p* Value
Heaviest lifetime BMI (kg/m^2^)	Category 1	48.1	0.99	0.6
	Category 2	49.3	1.4	
	Category 3	49.4	0.7	
Heaviest lifetime weight (kg)	Category 1	138.7	3.1	0.8
	Category 2	141.9	5.2	
	Category 3	140.9	2.3	
Onset of obesity (years)	Category 1	13.9	1.5	0.3
	Category 2	14	2.7	
	Category 3	11.5	0.9	
Age of hyperphagia (years)	Category 1	7.9	0.8	0.3
	Category 2	6.4	0.8	
	Category 3	6.9	0.4	
Positive history of childhood obesity	Category 1	57/83	23.9%	0.3
	Category 2	15/24	6.3%	
	Category 3	99/131	41.6%	
Positive history of hyperphagia during childhood	Category 1	68/83	28.6%	0.4
	Category 2	22/24	9.2%	
	Category 3	104/131	43.7%	
Positive family history of obesity	Category 1	67/83	28.2%	0.8
	Category 2	19/24	7.9%	
	Category 3	101/131	42.4%	

^1^ Category 1 (*n* = 83): genetic variants that have been approved for treatment with the melanocortin-4 receptor agonist (MC4R) a setmelanotide in Europe, as well as those under investigation for setmelanotide efficacy in clinical trials; category 2 (*n* = 24): genetic variants that may contribute to a diagnosis of genetic obesity but not a part of category 1; and category 3 (*n* = 131): no gene mutations. SEM: standard error of the mean.

## Data Availability

Some or all datasets generated during and/or analyzed during the current study are not publicly available but are available from the corresponding author on reasonable request.

## Supplementary Materials

The following supporting information can be downloaded at: https://www.mdpi.com/article/10.3390/jcm12196396/s1. Table S1: List of genes included in each category. Table S2: The genetic panel and risk assessments. Table S3: The prevalence of each genetic variant of uncertain significance among the positive cases.
