# Supplementary material for: Obesity Characteristics Are Poor Predictors of Genetic Mutations Associated with Obesity
Source: J Clin Med. 2023 Oct 7;12(19):6396. doi: 10.3390/jcm12196396 (PMC10573901; doi:10.3390/jcm12196396)
Supplement: Supplementary file 1 [file jcm-12-06396-s001.zip › jcm-2584407-supplementary.pdf]

Table S1. List of genes included in each category

**Category 1:** genetic variants that have been approved for treatment with the MC4R agonist setmelanotide in Europe, as well as those under investigation for setmelanotide efficacy in clinical trials.		
BBS1	*BBS20 (IFT172)*	*PHIP*
BBS2	*BBS21 (CFAP418)*	*PLXNA1*
BBS3 (ARL6)	*BBS22 (IFT74)*	*PLXNA2*
BBS4	*CPE*	*PLXNA3*
BBS5	*CREBBP*	*PLXNA4*
BBS6 (MKKS)	*DNMT3A*	*POMC*
BBS7	*HTR2C*	*RPGRIP1L*
BBS8 (TTC8)	*ISL1*	*SEMA3A*
BBS9 (PTHB1)	*KSR2*	*SEMA3B*
BBS10	*LEP*	*SEMA3C*
BBS11 (TRIM32)	*LEPR*	*SEMA3D*
BBS12	*MAGEL2*	*SEMA3E*
BBS13 (MKS1)	*MC3R*	*SEMA3F*
BBS14 (CEP290)	*MECP2*	*SEMA3G*
BBS15 (WDPCP)	*MRAP2*	*SH2B1*
BBS16 (SDCCAG8)	*NCOA1 (SRC1)*	*SIM1*
BBS17 (LZTFL1)	*NRP1*	*TBX3*
BBS18 (BBIP1)	*NRP2*	*TRPC5*
BBS19 (IFT27)	*PCSK1*	*TUB*
**Category 2:** genetic variants that may contribute to a diagnosis of genetic obesity but are neither currently indicated for setmelanotide treatment nor under investigation for setmelanotide efficacy in clinical trials.		
ADCY3	*GNAS*	*PPARG*
AFF4	*INPP5E*	*PROK2*
ALMS1	*KIDINS220*	*RAB23*
BDNF	*MC4R*	*RAI1*
CUL4B	*NR0B2*	*RPS6KA3*
DYRK1B	*NTRK2*	*UCP3*
EP300	*PCNT*	*VPS13B*
GNAS	*PHF6*	

Table S2. The genetic panel and risk assessments.

Gene	Gene name	Description (HGVS/ISCN)	Number detected	Zygosity	Classification (ACMG)
AFF4	ALF transcription elongation factor 4	NM_014423.4:c.455A>G p.(Asp152Gly)	1	Het.	VUS
ALMS1	ALMS1 centrosome and basal body associated protein	NM_015120.4:c.10775del p.(Thr3592Lysfs*6)	1	Het.	Pathogenic
		NM_001378454.1:c.3715_3718del p.(Ser1239fs)	1		Likely pathogenic
ARMC5	Armadillo repeat containing 5	NM_001105247.2:c.2597C>T p.(Pro866Leu)	1	Het.	VUS
BBS1	Bardet-Biedl syndrome 1	NM_024649.5:c.1169T>G p.(Met390Arg)	2	Het.	Pathogenic
		NM_024649.5:c.1169T>G p.(Met390Arg)	1		
BBS7	Bardet-Biedl syndrome 7	NM_176824.3:c.1458C>G p.(Tyr486*)	2	Het.	VUS
		NM_176824.3:c.1876dup p.(Ile626Asnfs*2)	2		
BBS9	Bardet-Biedl syndrome 9	NM_198428.3:c.(442+1_443-1)_(702+1_703-1)del	1	Het.	Pathogenic
CEP290	Centrosomal protein 290	NM_025114.4:c.4966G>T p.(Glu1656*)	1	Het.	Pathogenic
		C.(102+1_103-1)_(495+1_496-1)del	1		VUS
CREBBP	CREB binding protein	NM_004380.3:c.2980G>A p.(Val994Met)	1	Het.	VUS
		NM_004380.3:c.4372T>A p.(Leu1458Ile)	1		
CUL4B	Cullin 4B	NM_003588.3:c.52A>G p.(Thr18Ala)	1	Hem.	VUS
DNMT3A	DNA methyltransferase 3 alpha	NM_175629.2:c.58G>A p.(Glu20Lys)	1	Het.	VUS
		NM_175629.2:c.856-10G>A p.?	1		

DYRK1B	Dual specificity tyrosine phosphorylation regulated kinase 1B	NM_004714.3:c.106G>C p.(Ala36Pro)	1	Het.	VUS
EP300	E1A binding protein P300	NM_001429.4:c.2572A>G p.(Thr858Ala)	1	Het.	VUS
		NM_001429.4:c.3568A>G p.(Thr1190Ala)	1		
		NM_001429.4:c.902A>C p.(Asn301Thr)	1		
		NM_001429.4:c.952C>G p.(Pro318Ala)	1		
KIDINS220	Kinase D interacting substrate 220	NM_020738.4:c.4925G>A p.(Arg1642Gln)	1	Het.	VUS
		NM_020738.4:c.3617T>C p.(Leu1206Ser)	1		
		NM_020738.4:c.(?_1-1)_(108+1_109-1)del	1		
MAGEL2	MAGE family member L2	NM_019066.5:c.2635C>T p.(Pro879Ser)	1	Het.	VUS
		NM_019066.5:c.1375C>T p.(Arg459Cys)	1		
		NM_019066.5:c.2329C>A p.(Pro777Thr)	1		
		NM_019066.5:c.1586_1606del p.(Ala529_Gln535del)	1		
MC3R	Melanocortin-3 receptor	NM_019888.3:c.452G>A p.(Arg151His)	1	Het.	VUS
		NM_019888.3:c.770C>A p.(Pro257His)	1		
MC4R	Melanocortin-4 receptor	NM_005912.3:c.598A>G p.(Met200Val)	1	Het.	VUS
		NM_005912.3:c.89C>T p.(Ser30Phe)	1		
MECP2	Methyl CpG binding protein 2	NM_004992.3:c.605C>G p.(Ala202Gly)	1	Het.	VUS
		NM_004992.3:c.1102C>T p.(His368Tyr)	1	Hem.	

MRAP2	Melanocortin 2 receptor accessory protein 2	NM_138409.4:c.389A>G p.(Lys130Arg)	1	Het.	VUS
NCOA1	Nuclear receptor coactivator 1	NM_003743.5:c.2365A>G p.(Thr789Ala)	1	Het.	VUS
NRP1	Neuropilin 1	NM_003873.7:c.259G>A p.(Val87Met)	1	Het.	VUS
NRP2	Neuropilin 2	NM_201266.2:c.2173G>C p.(Gly725Arg)	1	Het.	VUS
NTRK2	Neurotrophic receptor tyrosine kinase 2	C.(2172+1_2173-1)_(*1_?)dup	1	Het.	VUS
		NM_006180.5:c.1345G>A p.(Val449Ile)	1		
		NM_006180.5:c.809A>G p.(Asn270Ser)	1		
PCNT	Pericentrin	NM_006031.6.c.362_363del p.(Thr121Serfs*3)	1	Het.	Likely pathogenic
		NM_006031.6:c.4090G>T p.(Glu1364*)	1		
		NM_006031.6:c.2347dup p.(Gln783Profs*8)	2		
PCSK1	Proprotein convertase subtilisin kexin type 1	NM_000439.5:c.661A>G p.(Asn221Asp)	13	Het.	VUS
		NM_000439.5:c.2129C>G p.(Pro710Arg)	1		
		NM_000439.5:c.1096A>G p.(Thr366Ala)	1		
		NM_000439.5:c.1030G>A p.(Ala344Thr)	1		
		NM_000439.5:c.375G>A p.(Met125Ile)	2		
		NM_000439.4:c.1918A>G p.(Thr640Ala)	3		
PHIP	Pleckstrin homology domain interacting protein	NM_017934.7:c.3670C>G p.(Leu1224Val)	1	Het.	VUS
		NM_017934.7:c.2579G>A p.(Gly860Glu)	1		

		NM_017934.7:c.3628A>G p.(Lys1210Glu)	1		
PLXNA1	Plexin A1	NM_032242.4:c.989T>C p.(Leu330Pro)	1	Het.	VUS
		NM_032242.4:c.1309G>C p.(Val437Leu)	1		
		NM_032242.4:c.211A>G p.(Ile71Val)	1		
PLXNA2	Plexin A2	NM_025179.4:c.973G>A p.(Ala325Thr)	1	Het.	VUS
		NM_025179.4:c.5243G>T p.(Trp1748Leu)	1		
		NM_025179.4:c.5503G>A p.(Glu1835Lys)	1		
		NM_025179.4:c.2983G>A p.(Glu995Lys)	2		
		NM_025179.4:c.95C>G p.(Ala32Gly)	1		
		NM_025179.4:c.1423G>T p.(Val475Leu)	1		
		NM_025179.4:c.5251G>A p.(Val1751Met)	1		
PLXNA3	Plexin A3	NM_017514.5:c.139G>A p.(Val47Met)	1	Het.	VUS
		NM_017514.5:c.1048C>T p.(Arg350Trp)	1		
		NM_017514.5:c.1423A>G p.(lle475Val)	1		
PLXNA4	Plexin A4	NM_020911.1:c.1259G>T p.(Arg420Leu)	1	Het.	VUS
		NM_020911.2:c.299T>A p.(Ile100Asn)	1		
		NM_020911.1:c.4903C>T p.(Arg1635Cys)	1		
		NM_020911.2:c.3492+3G >A p.?	1		
		NM_020911.2:c.1570G>A p.(Asp524Asn)	1		

POMC	Proopiomelanocortin	NM_001035256.2:c.706C>G p.(Arg236Gly)	5	Het.	Likely pathogenic
		NM_001035256.2:c.394C>G p. (Pro132Ala)	3		VUS
PROK2	Prokineticin 2	c.(222+1_223-1)_(*1_?)del	1	Het.	VUS
		NM_001126128.2:c.297dup p.(Gly100Trpfs*22)	1		
RAI1	Retinoic Acid Induced 1	NM_030665.4:c.867_872dup p.(Gln290_Gln291dup)	1	Het.	VUS
		NM_030665.4:c.194C>T p.(Thr65Met)	1		
		NM_030665.4:c.864_872dup p.(Gln289_Gln291dup)	1		
		NM_030665.4:c.4996C>G p.(Leu1666Val)	1		
RPGRIP1L	RPGRIP1 like	NM_015272.5:c.2200C>T p.(Arg734*)	1	Het.	Likely pathogenic
		NM_015272.5:c.1489G>T p.(Glu497*)	1		
RPS6KA3	Ribosomal protein S6 kinase A3	c.(774+1_775-1)_(1102+1_1103-1)del	1	Het.	Likely pathogenic
SDCCAG8	SHH signalling and ciliogenesis regulator SDCCAG8	NM_006642.5:c.696T>G p.(Tyr232*)	1	Het.	Likely pathogenic
SEMA3A	Semaphorin 3A	NM_006080.3:c.1799A>T p.(Lys600Met)	1	Het.	VUS
		NM_006080.3:c.458A>G p.(Asn153Ser)	1		
SEMA3B	Semaphorin 3B	NM_004636.2:c.1860G>C p.(Glu620Asp)	1	Het.	VUS
		NM_004636.2:c.586C>T p.(Arg196*)	1		
SEMA3C	Semaphorin 3C	NM_006379.5:c.857C>T p.(Ala286Val)	1	Het.	VUS
SEMA3D	Semaphorin 3D	NM_152754.3:c.113T>C p.(Leu38Ser)	1	Het.	VUS

SEMA3 E	Semaphorin 3E	NM_012431.3: c.283T>Gp.(Trp95Gly)	1	Het.	VUS
		NM_012431.3:c.214G>T p.(Val72Leu)	1		
		NM_012431.3:c.(115+1_1 16-1)_(336+1_337-1)del	1		
		NM_012431.3:c.1514T>C p.(Ile505Thr)	1		
SEMA3 F	Semaphorin 3F	NM_004186.5:c.533C>T p.(Pro178Leu)	1	Het.	VUS
		NM_004186.5:c.1352G>A p.(Arg451GIn)	1		
		NM_004186.5:c.1588-3C>T p.?	1		
SEMA3 G	Semaphorin 3G	NM_020163.3:c.200G>A p.(Arg67Gln)	1	Het.	VUS
		NM_020163.3:c.1574dup p.(Tyr525*)	1		
SH2B1	SH2B adaptor protein 1	NM_001145795.2:c.2083 G>A p.(Val695Met)	1	Het.	VUS
SIM1	Single-minded homolog 1	NM_005068.3:c.390G>A p.(Pro130=)	2	Het.	VUS
		NM_005068.3:c.1720A>G p.(Met574Val)	1		
		NM_005068.3:c.2135C>T p.(Thr712Ile)	1		
		NM_005068.3:c.452T>G p.(Val151Gly)	1		
		NM_005068.3:c.383T>C p.(Ile128Thr)	1		
		NM_005068.3:c.1445C>G p.(Pro482Arg)	2		
TRPC5	Transient receptor potential channel 5	NM_012471.3:c.2212C>A p.(Leu738Ile)	3	Het.	VUS
UCP3	Uncoupling protein 3	NM_003356.4:c.370T>A p.(Cys124Ser)	1	Het.	VUS

		NM_003356.4:c.304G>A p.(Val102Ile)	1		
		NM_003356.4:c.88G>A p.(Val30Ile)	1		
VPS13B	Vacuolar protein sorting 13 homolog B	NM_017890.4:c.11695_11698del p.(Ser3901Argfs*40)	1	Het.	Likely pathogenic
		NM_017890.4:c.8172+1G>A p.?	1		

HGVS/ISCN: Human Genome Variation Society/International System for human Cytogenetic Nomenclature; ACMG: American College of Medical Genetics and Genomics; VUS: variant of uncertain significance; Het: heterozygous; Hem: hemizygous.

Table S3. The prevalence of each genetic variant of uncertain significance among the positive cases.

Gene	Zygosity	Number detected	Percentage (%)
AFF4	Het.	1	0.7
ARMC5	Het.	1	0.7
BBS7	Het.	4	2.8
CEP290	Het.	1	0.7
CREBBP	Het.	2	1.4
CUL4B	Hem.	1	0.7
DNMT3A	Het.	2	1.4
DYRK1B	Het.	1	0.7
EP300	Het.	4	2.8
KIDINS220	Het.	3	2.1
MAGEL2	Het.	4	2.8
MC3R	Het.	2	1.4
MC4R	Het.	2	1.4
MECP2	Het.	1	0.7
	Hem.	1	0.7
MRAP2	Het.	1	0.7
NCOA1	Het.	1	0.7

NRP1	Het.	1	0.7
NRP2	Het.	1	0.7
NTRK2	Het.	3	2.1
PCSK1	Het.	21	14.9
PHIP	Het.	3	2.1
PLXNA1	Het.	3	2.1
PLXNA2	Het.	8	5.7
PLXNA3	Het.	3	2.1
PLXNA4	Het.	5	3.5
POMC	Het.	3	2.1
PROK2	Het.	2	1.4
RAI1	Het.	4	2.8
SEMA3A	Het.	2	1.4
SEMA3B	Het.	2	1.4
SEMA3C	Het.	1	0.7
SEMA3D	Het.	1	0.7
SEMA3E	Het.	4	2.8
SEMA3F	Het.	3	2.1
SEMA3G	Het.	2	1.4

SH2B1	Het.	1	0.7
SIM1	Het.	8	5.7
TRPC5	Het.	3	2.1
UCP3	Het.	3	2.1

Het: heterozygous; Hem: hemizygous.
